# Molluscicidal Activity of Extracts and Fractions From *Hagenia abyssinica*, *Rosa abyssinica*, and *Cucumis ficifolius* Against *Biomphalaria* and *Bulinus* Snails

**DOI:** 10.1155/japr/7968654

**Published:** 2024-11-25

**Authors:** Hirut Basha, Asfaw Debella, Milkyas Endale, Eyob Debebe, Meharu Mathewos, Tesfaye Biftu, Hassen Mamo

**Affiliations:** ^1^Traditional and Modern Medicine Research and Development Directorate, Armauer Hansen Research Institute, PO Box 1005, Addis Ababa, Ethiopia; ^2^Biomedical Engineering, 503 Cantor Trail, Cherry Hill, New Jersey 08002, USA; ^3^Department of Microbial, Cellular and Molecular Biology, College of Natural and Computational Sciences, Addis Ababa University, PO Box 1176, Addis Ababa, Ethiopia

**Keywords:** *Biomphalaria* and *Bulinus* snails, *Hagenia abyssinica*, molluscicidal, schistosomiasis

## Abstract

**Background**: Schistosomiasis continues to be a major public health concern in Ethiopia. Eliminating the intermediate host snails is an effective and cost-efficient strategy for preventing and controlling schistosomiasis transmission. However, chemical molluscicides have limitations due to their toxicity to nontarget aquatic organisms, environmental concerns, and the development of resistance. Plant-based molluscicides are biodegradable, less toxic, safe, and cost-effective.

**Objective**: This study is aimed at evaluating the molluscicidal activity of *Hagenia abyssinica* flowers, *Rosa abyssinica* fruits, and *Cucumis ficifolius* roots against *Biomphalaria* and *Bulinus* species.

**Methods**: Adult *Biomphalaria* and *Bulinus* species were subjected to varying concentrations of aqueous and 70% ethanol extracts and solvent partitions from *H. abyssinica*, *R. abyssinica*, and *C. ficifolius* for 24, 48, and 72 h. The investigation involved conducting a phytochemical analysis using standard screening methods. Female mice were subjected to an acute oral toxicity test using a 70% ethanol extract of *H. abyssinica*, *R. abyssinica*, and *C. ficifolius*. The mortality data were then determined using GraphPad Prism 9 software.

**Results**: Aqueous and 70% ethanol extracts of *R. abyssinica* and *C. ficifolius* did not exhibit molluscicidal activities against *Biomphalaria* and *Bulinus* species. However, aqueous, 70% ethanol, and chloroform extracts of *H. abyssinica* showed significant molluscicidal activities against *Biomphalaria* species with 24-h LC_50_ values of 39.05, 11.93, and 5.52 mg/L, respectively. Similarly, the LC_50_ values of the same extracts against *Bulinus* species after 24 h of exposure were 40.08, 12.23, and 6.13 mg/L, respectively. The plant extract's LD_50_ for acute toxicity against mice was found to be over 2000 mg/kg of body weight.

**Conclusion**: *H. abyssinica* demonstrated potent molluscicidal activity, making it a potential candidate for application. Further isolation of active ingredients and field trials are necessary to determine the optimal conditions for its use in snail control.

## 1. Background

Schistosomiasis, a neglected tropical disease, affects around 240 million individuals globally, with over 700 million at risk in tropical and subtropical regions [1, 2]. Sub-Saharan Africa bears the brunt, representing 80% of global cases [3]. Ethiopia is one such country grappling with this disease, with an estimated 5 million infections and over 37.5 million people at risk [4]. The two most common species in Ethiopia are *Schistosoma mansoni* and *Schistosoma haematobium*. They are spread by *Biomphalaria* and *Bulinus* snails, respectively [5]. Though *S. mansoni* is widespread across the country, *S. haematobium* is confined to a few isolated areas [6].

Schistosomiasis remains a formidable public health challenge in Ethiopia due to multiple factors. Widespread open defecation practices contribute to the contamination of water sources with schistosomiasis eggs [7]. Moreover, a significant proportion of the population depends on unimproved water sources such as rivers, ponds, and lakes for bathing and drinking, elevating the risk of schistosomiasis transmission [8]. The proliferation of irrigation projects, hydroelectric dam constructions, and water conservation initiatives provide conducive environments for parasite development [9].

Efforts to prevent and control schistosomiasis encompass various strategies, including early diagnosis and treatment, health education initiatives, ensuring access to safe water sources, improving sanitation coverage, and eliminating the intermediate host snails [10, 11]. Targeting these snail hosts represents a cost-effective approach to controlling transmission [12, 13]. Plant-derived molluscicides offer several advantageous characteristics, including easy biodegradability, lower toxicity, safety in use, and cost-effectiveness. In contrast, chemical molluscicides pose limitations due to their toxicity to nontarget aquatic organisms, environmental concerns, and the development of resistance [14–16].

Numerous studies have highlighted the medicinal potential of certain plants in controlling *Schistosoma* snail intermediate hosts [17–21]. This study is aimed at evaluating the molluscicidal properties of *Hagenia abyssinica* flowers, *Rosa abyssinica* fruits, and *Cucumis ficifolius* roots against *Biomphalaria* and *Bulinus* species. These plants are commonly utilized by traditional healers and the community for treating various ailments [22–25]. *H. abyssinica* flower is known for its efficacy against parasites, especially tapeworm infections [25]. *C. ficifolius* is traditionally used for treating liver disease and bloody diarrhea, among other ailments [26], while *R. abyssinica* is employed in managing chronic conditions such as diabetes, hypertension, and rheumatic pain [27]. Investigating these plants' potential as molluscicides offers an opportunity to explore alternative, environmentally friendly methods for controlling schistosomiasis.

## 2. Materials and Methods

### 2.1. Plant Material Collection and Authentication

*R. abyssinica* fruits and *C. ficifolius* roots were collected from Ensaro Woreda, Semien Shewa zone, Amhara Regional State, Ethiopia. The coordinates of the collection sites are between 9°35⁣′−9°55⁣′ N and 38°50⁣′−39°5⁣′ E. The female *H. abyssinica* flowers were collected from Gulele Botanical Garden, Addis Ababa, Ethiopia. The garden's geographical coordinates are 9.07879504343465 and 38.721098843590156. Mr. Melaku Wondafrash, a botanist affiliated with Addis Ababa University, verified the gathered plant specimens. The National Herbarium of Addis Ababa University holds voucher specimens marked with HB002, HB006, and HB005, which are associated with *H.* abyssinica, *R. abyssinica*, and *C. ficifolius*, respectively.

*H. abyssinica* (Bruce) J. F. Gmel., called Kousso or Kosso in East and Central Africa, is the only species in the genus *Hagenia* (Rosaceae) [28]. This tree, which is dioecious, is a distinctive plant found in the Afro-Montane Forests of Central and Eastern Africa [28]. *H. abyssinica* typically prospers at elevations ranging from 2000 to 3000 m annually [29]. *C. ficifolius*, conversely, is located at altitudes between 1070 and 2700 m [30]. *R. abyssinica* plants thrive at elevations between 1900 and 2050 m and are capable of adjusting to areas with varying levels of precipitation [31].

### 2.2. Plant Part Preparation

The collected plant samples were cleaned to eliminate any dirt before drying and processing. After the plants were dried, they were pulverized with a grinder and then filtered through a 30–40-mm mesh, which was found to be the best size for extraction.

### 2.3. Plant Extraction

#### 2.3.1. Hydroalcoholic Extraction

For the hydroalcoholic extraction, 100 g of *H. abyssinica* flowers, 120 g of *R. abyssinica* fruits, and 150 g of *C. ficifolius* roots were placed in separate Erlenmeyer flasks. Each powdered plant material was soaked in 70% ethanol and shaken on an orbital shaker (Benchmark Scientific, Inc. United States) for 24 h to enhance extraction.

The solution was passed through Whatman No. One filter paper to separate out any solid particles. The remaining substance underwent three additional macerations, each lasting 24 h, and the resulting solvents were removed using a rotary evaporator operating at 40°C (BUCHI Labortechnik AG, Switzerland).

The remaining solution was transferred to a container and placed in a Daihan Digital General Purpose water bath (DAIHAN Scientific Co., South Korea) set at a temperature of 40°C for approximately 2–3 days to remove any leftover alcohol and dry the extracts. The dried extracts were then kept in sealed glass containers in a refrigerator until they were used for the experiment. The yield of the extracts was 11.4 g from *H. abyssinica* flowers, 13.9 g from *R. abyssinica* fruits, and 6.4 g from *C. ficifolius* roots [32].

#### 2.3.2. Solvent Partition/Fractionation of the Hydroalcoholic Extract

The 70% ethanol extract of *H. abyssinica* was separated further using the organic solvent chloroform and ethyl acetate. To begin, the initial 70% ethanol extract was either dissolved or suspended in a small amount of water and then transferred to a separatory funnel. It was then defatted by adding 30 mL of petroleum ether (40°C–60°C) three times, shaking until the petroleum ether lost its color. The petroleum ether layer was then separated, dried, and stored separately. Subsequently, chloroform (50 mL, added three times) was introduced to the separatory funnel containing the defatted crude extract. The partitioning process with chloroform was continued until the chloroform layer became colorless.

The fraction of chloroform was separated, dried using a rotary evaporator, and stored separately. The aqueous residue of the raw extract was partitioned similarly with ethyl acetate as previously described. The dried ethyl acetate fraction was stored separately and designated as the ethyl acetate fraction. The remaining aqueous residue was dried and designated as the aqueous residue [33–35].

#### 2.3.3. Aqueous Extraction

To prepare the aqueous extract, 100 g of *H. abyssinica* flowers, 100 g of *R. abyssinica* fruits, and 100 g of *C. ficifolius* roots were separately placed in Erlenmeyer flasks and soaked in distilled water. The flasks were then continuously agitated on an orbital shaker for 6 h, with occasional filtering of the macerate every 2 h, followed by the addition of more distilled water to maximize extraction. The resulting aqueous macerates were filtered through muslin gauze, and the filtrate was subsequently dried using a lyophilizer (LYOQUEST-55 PLUS, Spain). The resulting lyophilized dry powders were collected in screw-capped glass containers, weighed, and stored in desiccators to prevent moisture absorption until used for the experiments.

### 2.4. Phytochemical Screening

The extracts were subjected to phytochemical analysis to detect the existence of diverse phytochemical components, including alkaloids, flavonoids, tannins, saponins, phenols, and terpenoids. Each phytochemical screening test was conducted following standard protocols [36].

### 2.5. Test for Alkaloids (Dragendorff's Test)

When 2 mL of the extract was mixed with 1 mL of Dragendorff's reagent, it caused the formation of an orange–red solid, indicating the existence of alkaloids.

### 2.6. Test for Flavonoids (Alkaline Reagent Test)

Two to three drops of sodium hydroxide were added to 2 mL of the solution, which produced a deep yellow hue. After adding a small amount of diluted hydrochloric acid, the color slowly faded and turned colorless, suggesting the existence of flavonoids.

### 2.7. Test for Phenol and Tannins (Ferric Chloride Test)

One milliliter of the extract was mixed with 2 mL of a 5% neutral ferric chloride solution. This caused the mixture to turn dark blue, which indicated that phenolic compounds and tannins are present.

### 2.8. Test for Saponins (Foam Test)

One milliliter of the extract solution was mixed with distilled water to make a total volume of 20 mL. Then, the mixture was shaken in a graduated cylinder for 15 min. The formation of long-lasting foam indicated the existence of saponins.

### 2.9. Test for Steroids (Salkowski Test)

The sample was mixed with chloroform, followed by the addition of concentrated H_2_SO_4_ on the walls of the test tube. The development of a red color confirmed the existence of steroids.

### 2.10. Tests for Terpenoids (Salkowski Test)

A mixture was prepared by combining 5 mL of the extract with 2 mL of chloroform, followed by the careful addition of 3 mL of concentrated sulfuric acid to form a layer. The appearance of a reddish–brown color at the boundary confirmed the existence of terpenoids.

### 2.11. Acute Toxicity

The study used female Swiss Albino mice (25–30 g) obtained from the breeding center of the Ethiopian Public Health Institute. After acclimating to the laboratory conditions for 1 week, the mice were individually housed in standard aluminum cages, maintained at a temperature of 20°C ± 2°C, a relative humidity of 65% ± 0.5%, and a 12-h light–dark cycle. They were provided with a standard commercial diet and access to clean tap water throughout the experiment. All animals involved in this study were treated humanely throughout the study period in accordance with the International Guidelines of Laboratory Animal Care and Use [37].

An experiment was conducted on female mice to evaluate the acute oral toxicity of 70% ethanol, aqueous, and chloroform fraction extracts from *H. abyssinica* flowers, following the Organisation for Economic Co-operation and Development 423 guidelines [38]. Forty-five female mice were divided into 15 groups, each containing three mice. The first five groups (Groups I–V) were given the 70% ethanol extracts of *H. abyssinica*, Groups VI–X received the aqueous extracts, and the remaining five groups (Groups XI–XV) were given the chloroform fraction extracts of *H. abyssinica*.

Groups I, VI, and XI served as the negative control for 70% ethanol, aqueous, and chloroform fraction extracts of *H. abyssinica* flower, respectively, and received a vehicle. Groups II–V, Groups VII–X, and Groups XII–XV received a single dose of 70% ethanol, aqueous, and chloroform fraction extracts of *H. abyssinica* flower at varying concentrations of 5, 50, 300, and 2000 mg/kg, respectively, after fasting for 3–4 h. The extract was administered using an oral gavage feeding needle. After administering the extract, food could be withheld for an additional 1–2 h. The animals were then individually monitored for signs of toxicity starting from 30 min after treatment up to 24 h. Behavioral signs of toxic manifestations such as piloerection, debilitation, tremors, excitability, salivation, twitching, diarrhea, and lethargy were observed and recorded until the end of the 14 days [38]. For euthanizing the animal, pentobarbital 150 mg/kg IP was used as suggested by the American Veterinary Medical Association (AVMA) guidelines [39]. This study was performed following the ARRIVE guidelines for reporting animal research [40].

### 2.12. Snail Collection and Maintenance

Mature *Biomphalaria* species were gathered from Tikur Wuha, located within the geographic coordinates ranging from 6°04⁣′00⁣^″^ to 7°10⁣′00⁣^″^ N latitude and 38°26⁣′30⁣^″^ to 38°43⁣′00⁣^″^ E longitude. Adult *Bulinus* snails were obtained from the Alwero reservoir in Abobo, Gambella Region, situated 822 km southwest of Addis Ababa, at the geographical coordinates of 7°52⁣′19.2⁣^″^ N and 34°29⁣′56.4⁣^″^ E latitude and longitude, respectively. Using dip-net scoops, the snails were retrieved from the water, cleaned to remove any attached debris, and then placed in open plastic buckets containing vegetation and water. The collected snails were then transported to the laboratory of the Traditional and Modern Medicine Research and Development Directorate, Armauer Hansen Research Institute. Standard identification keys were used to identify and authenticate the snails, a task overseen by Professor Seid Tiku from Jimma University.

The snails that were collected were kept in aquaria at temperatures ranging from 25°C to 28°C for 1–2 weeks to get used to the lab conditions. To check for cercarial infection, the snails were exposed to artificial light for 1–2 h to induce cercarial shedding. Any snails showing signs of patent trematode infections were not used in the experiments. The snails were given oven-dried lettuce leaves every 24 h, and the water in the tanks was changed every 3 days. Each test was repeated three times independently [41, 42].

### 2.13. Preparation of Niclosamide (Standard Drugs Used as a Positive Control)

A powder formulation of niclosamide (70% WP) was purchased from APExBIO Technology LLC America. According to the WHO's 2019 guidelines, niclosamide 70% WP 1 mg/L was utilized as a positive control [41].

### 2.14. Molluscicidal Activity Test and Preparation of the Stock Solution

The experiments were conducted in accordance with the 2019 WHO guidelines and standard test procedures for assessing molluscicidal plants [41].

Initially, a 100 mg/L stock solution was prepared, and then, various working solutions were serially diluted from this stock solution for the experiment. These working solutions included 80, 60, 40, 30, 15, 5, 1, and 0.5 mg/L. These solutions were prepared to test molluscicidal activity against *Biomphalaria* and *Bulinus* species.

Glass containers of water containing groups of 10 snails of comparable sizes, 9–11-mm *Biomphalaria* and 5–7-mm *Bulinus*, were used for the tests with the test substances. A preliminary test was carried out to establish the most suitable concentration range. The plant extracts were diluted to the necessary concentrations using distilled water. The experiments were repeated three times. Distilled water was used as a control for aqueous and 70% ethanol extraction, while 2% Tween 80 served as a control for solvent fractionation. Distilled water and 2% Tween 80 were utilized as media for dissolving the extracts. All 10 snails were submerged in the solution at room temperature for 24, 48, and 72 h.

The suspension was poured off after 24 h, and the snails were rinsed four times with aged water (water without chlorine) and then placed in extract-free aged water for an additional 24-h recovery period. Snail mortality was assessed by observing the lack of movement, the inability of the snail's flesh to retract into the shell when mechanically stimulated (probed), and the presence of bleeding [20]. If more than 10% of the snails in the negative control group died, the replicate control test was invalidated, and new replicate tests were conducted [41].

### 2.15. Statistical Analysis

The mortality data were analyzed using GraphPad Prism 9. The LC_50_ and LC_90_ represent the concentrations needed to kill 50% and 90% of the test organisms. The analysis used a significance level below 0.05 and a 95% confidence interval (CI).

## 3. Results

### 3.1. Acute Toxicity Study

The experiment on acute toxicity showed that the test animals did not exhibit any signs of toxicity or noticeable changes in behavior when given the highest oral dosage of 2000 mg/kg of the 70% ethanol, aqueous, and chloroform fraction extracts of *H. abyssinica* flowers. Furthermore, no fatalities occurred during the 14-day monitoring period. These results indicated that the lethal dose (LD_50_) of the 70% ethanol, aqueous, and chloroform fraction extracts of *H. abyssinica* flowers exceeds 2000 mg/kg of body weight.

### 3.2. Phytochemical Analysis

The aqueous extract of *H. abyssinica* flowers was found to contain saponins, steroids, terpenoids, alkaloids, phenols, tannins, and flavonoids according to the phytochemical screening test (Table 1). Similarly, the phytochemical analysis of the aqueous extract derived from *R. abyssinica* fruit indicated the presence of terpenoids, steroids, phenols, tannins, glycosides, saponins, and flavonoids. The aqueous extracts from the roots of *C. ficifolius* were analyzed for phytochemicals and were found to contain flavonoids, glycosides, saponins, and alkaloids.

### 3.3. Molluscicidal Activities

The study found that the aqueous and 70% ethanol extracts from *R. abyssinica* fruits and *C. ficifolius* roots did not show any molluscicidal effects on *Biomphalaria* and *Bulinus* species. Furthermore, the ethyl acetate fraction and water residue from *H. abyssinica* flowers also did not exhibit any molluscicidal activity. However, the aqueous, 70% ethanol, and chloroform fraction extracts from *H. abyssinica* flowers demonstrated molluscicidal activities against *Biomphalaria* and *Bulinus* species (Table 2).

The 24-h LC_50_ values for the aqueous, 70% ethanol, and chloroform fraction extracts from *H. abyssinica* flowers against *Biomphalaria* species were 39.05, 11.93, and 5.52 mg/L, respectively (Table 2). Similarly, the LC_50_ values of the same extracts against *Bulinus* species after 24 h of exposure were 40.08, 12.23, and 6.13 mg/L, respectively. The results indicated that the 24-h LC_90_ values of the aqueous, 70% ethanol, and chloroform fraction extracts from *H. abyssinica* flowers against *Biomphalaria* were 65.38, 26.64, and 13.65 mg/L, respectively. Likewise, the LC_90_ values of the same extracts against *Bulinus* species after 24 h of exposure were 74.4, 28.57, and 17.5 mg/L, respectively (Table 2).

Comparing the LC_50_ and LC_90_ values of the various extracts revealed that the chloroform fraction demonstrated the highest molluscicidal activity, with the 70% ethanol extract following closely behind as indicated in Table 2.

The positive control niclosamide had an LC_50_ of 0.37 and 0.41 mg/L for *Biomphalaria* and *Bulinus* snails, respectively.

**Figure 1:**
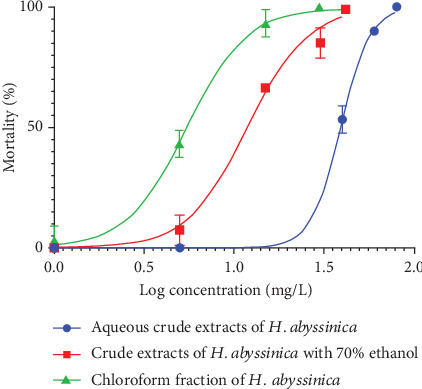
*Biomphalaria* species mortality (percent) versus the 24-h base logarithm of the concentration (milligrams per liter): aqueous, 70% ethanol, and chloroform fraction extracts of the *H. abyssinica* flower.

The flower extracts of *H. abyssinica* exhibited molluscicidal activity against *Biomphalaria* in a dose-dependent manner. The mortality of the snails tended to rise as the concentration of the extracts increased, as shown in Figure 1.

**Figure 2:**
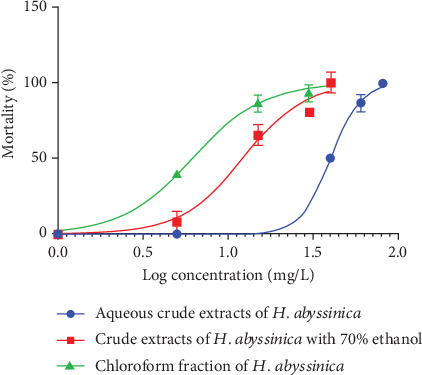
*Bulinus* species mortality (percent) versus the 24-h base logarithm of the concentration (milligrams per liter): aqueous, 70% ethanol, and chloroform fraction extracts of the *H. abyssinica* flower.

Similarly, the aqueous, 70% ethanol, and chloroform fraction extracts of the *H. abyssinica* flower demonstrated dose-dependent molluscicidal activity against *Bulinus*. The mortality rate of the snails rose as the concentration of the extracts increased, as shown in Figure 2.

## 4. Discussion

This study examined the molluscicidal effects of the aqueous, 70% ethanol, and chloroform fraction extracts of *H. abyssinica* flower against *Biomphalaria* and *Bulinus* species. The activities were found to be time- and concentration-dependent. However, the fruit of *R. abyssinica* and the root of *C. ficifolius*, when prepared using water and 70% ethanol, did not exhibit molluscicidal activities against *Biomphalaria* and *Bulinus* species. The molluscicidal effect of *H. abyssinica* flower against *Biomphalaria* and *Bulinus* species is being reported herein for the first time. The potency of the aqueous extract of *H. abyssinica* flower in this study was higher than previous studies conducted on *Glinus lotoides*, *Balanites aegyptiaca*, and *Achyranthes aspera* as molluscicidal plants [20, 42, 43]. Kiros et al. reported an LC_50_ of 47.1 mg/L for the *G. lotoides* aqueous extract on *Biomphalaria pfeifferi* after 24 h [20]. The study conducted by Mandefro et al. on the *A. aspera* aqueous leaf extract found an LC_50_ of 72.4 mg/L after 24 h [43]. The ethanol extract of *H. abyssinica* flower had a greater potency than the aqueous extract, as demonstrated by the comparison of LC_50_ and LC_90_ values. This finding is consistent with other research that assessed the aqueous and ethanolic extracts of *Dalbergia* sissoo plant parts on *B. pfeifferi* [44]. The findings were also consistent with those of Otarigho and Morenikeji, who found comparable outcomes while assessing the effectiveness of water-based and alcohol-based leaf extracts of *Chromolaena odorata* as molluscicides [45]. The variation in activity levels between the two extracts could be due to the choice of solvent or the extraction method [45]. Additionally, upon comparing the LC_50_ and LC_90_ values of the chloroform fraction extracts from the flower of *H. abyssinica*, it was found that it exhibited greater potency than the crude aqueous and ethanol extracts from *H. abyssinica*.

This finding aligns with a study on the molluscicidal activity of *Schinopsis brasiliensis* stem bark [46]. The chloroform extract may be effective due to the solvent's ability to extract nonpolar active compounds, such as phenols, flavonoids, tannins, and triterpenes [33, 47]. Some of these active compounds, such as flavonoids and triterpenes, are known to have molluscicidal activity, making chloroform extract more potent than aqueous and 70% ethanol extracts [48–50].

Phytochemical analysis of the aqueous extract of *H. abyssinica* flower in this study indicated the presence of saponins, steroids, terpenoids, alkaloids, phenols, tannins, and flavonoids. The molluscicidal activity of the flower *H. abyssinica* can be attributed to the presence of these compounds either together or independently [20]. Several studies have indicated that saponins are one of the major classes of compounds possessing molluscicidal activity [20, 43, 51, 52]. The mechanism of action of saponin compounds has been well documented in several studies, showing that they cause damage to the cell membrane, affecting the snails' cellular permeability and osmoregulatory functions [20, 53]. Other important compounds found in this plant include flavonoids, alkaloids, and terpenoids which are known for their molluscicidal properties. The alkaloids and terpenoids can cause neurotoxicity in snails by acting as acetylcholinesterase inhibitors [53, 54].

The phytochemical analysis in this study also revealed the presence of saponins in the root of *C. ficifolius* and the fruit of *R. abyssinica* extracts. However, these extracts did not show molluscicidal activities against *Biomphalaria* and *Bulinus* species. This suggests that not all types of saponins may possess the expected molluscicidal activities. Their activities depend on the chemical structure, including the number of sugar moieties and their location in the aglycone portion of the structure [54]. Also, the synergistic effect of the components that exist in *H. abyssinica* might be a reason for its noticeable effect.

The results of this study revealed that as the exposure time extended from 24 to 72 h, the LC_50_ and LC_90_ of aqueous, ethanol, and chloroform fraction extracts of *H. abyssinica* flower against both *Biomphalaria* and *Bulinus* species decreased. This decrease may be due to the slow release of the active ingredient in the plant product, which could have a gradual influence on the snails' survival [43].

In the present study, the 24-h LC_50_ values of the aqueous, 70% ethanol, and chloroform fraction extracts of *H. abyssinica* flower against *Biomphalaria* were below 40 mg/L (i.e., 39.05, 11.93, and 5.52 mg/L, respectively). According to WHO (1993) guidelines, plant extracts with LD_50_ values below 40 mg/L can be directly employed against mollusk populations [55]. In line with the 2019 WHO guidelines, a plant can be classified as a potential molluscicide if it can eliminate 90% of snails within 24 h of exposure without surpassing the concentration of 100 mg/L [41]. In this study, the 24-h LC_90_ values of the aqueous, 70% ethanol, and chloroform fraction extracts of *H. abyssinica* flower against *Biomphalaria* species were 65.38, 26.64, and 13.65 mg/L, respectively. Likewise, the 24-h LC_90_ of the same extracts against *Bulinus* species was 74.4, 28.57, and 17.5 mg/L, respectively. Therefore, the aqueous, 70% ethanol, and chloroform fraction extracts of *H. abyssinica* flower can be considered molluscicide candidates and require further evaluation through field trials.

## 5. Conclusion

This study highlighted the potential molluscicidal activity of *H. abyssinica* flower extracts as effective molluscicides against *Biomphalaria* and *Bulinus* species, which are intermediate hosts for schistosomiasis. The extracts exhibited time- and concentration-dependent activities, with the chloroform fraction extract showing the highest potency compared to 70% ethanol and aqueous extracts. The flower extract's inclusion of saponins, alkaloids, terpenoids, flavonoids, and other compounds implies their role in the detected molluscicidal effects. The use of these extracts in screening for plant molluscicides, according to WHO guidelines, could contribute to the range of approaches available for managing snails that transmit schistosomiasis in tropical and Third World countries where the disease is prevalent. The findings support further exploration of *H. abyssinica* flower extracts as potential environmentally friendly alternatives for controlling schistosomiasis transmission, warranting field trials to validate their effectiveness. Further research is needed to identify and isolate the specific classes of saponins that exhibit molluscicidal effects.

## Figures and Tables

**Table 1:** Phytochemical screening of *H. abyssinica* flower, *R. abyssinica* fruit, and *C. ficifolius* root extracts.

**Plants**	**Solvents**	**Alkaloids**	**Flavonoids**	**Phenols and tannins**	**Saponins**	**Steroids**	**Terpenoids**
*H. abyssinica*	Aqueous	+	+	+	+	+	+
	70% ethanol	+	+	+	+	+	+
	Chloroform	+	+	+	+	+	+

*R. abyssinica*	Aqueous	−	+	+	+	+	+
	70% ethanol	−	+	+	+	−	−

*C. ficifolius*	Aqueous	+	+	−	+	−	−
	70% ethanol	+	+	−	+	−	−

*Note:* +: present; −: absent.

**Table 2:** Molluscicidal effect of the crude extract and solvent fractions of *H. abyssinica* fruit against *Biomphalaria* and *Bulinus* species.

**Snail species**	**Exposure hours**	***Hagenia abyssinica* flower**					
		**Aqueous extract**		**70% ethanol extract**		**Chloroform fraction**	
		**L** **C**_50_ ** (mg/L) (CI)**	**L** **C**_90_ ** (mg/L) (CI)**	**L** **C**_50_ ** (mg/L) (CI)**	**L** **C**_90_ ** (mg/L) (CI)**	**L** **C**_50_ ** (mg/L) (CI)**	**L** **C**_90_ ** (mg/L) (CI)**
*Biomphalaria*	24 h	39.0 (38.13–39.87)	65.3 (57.20–78.93)	11.9 (10.86–13.04)	26.6 (20.28–41.57)	5.5 (5.15–5.92)	13.6 (8.93–19.94)
	48 h	39.1 (38.12–40.07)	57.8 (48.81–70.48)	11.8 (10.44–13.37)	25.1 (18.00–48.25)	5.5 (5.15–5.92)	13.6 (8.93–19.94)
	72 h	39.1 (38.12–40.07)	57.4 (48.81–70.48)	10.8 (9.72–11.98	19.9 (16.00–26.7)	4.7 (4.33–5.07)	13.4 (8.81–20.8)

*Bulinus*	24 h	40.0 (39.05–41.04)	74.4 (63.75–94.35)	12.2 (10.78–13.76)	28.5 (19.37–67.81)	6.1 (5.68–6.61)	17.5 (12.36–26.52)
	48 h	39.0 (38.13–39.87)	65.8 (57.2–78.93)	12.0 (10.74–13.4)	26.6 (19.16–49.94)	5.4 (4.97–6.04)	17.3 (10.95–31.54)
	72 h	39.0 (38.13–39.87)	65.8 (57.2–78.93)	12.0 (10.74–13.4)	26.6 (19.16–49.94)	5.3 (4.88–5.83)	15.7 (10.11–26.41)

*Note:* The distilled water (negative control) had zero mortality for both snail species.

Abbreviation: CI, confidence interval.

## Data Availability

The data that support the findings of this study are available from the corresponding author upon reasonable request.
